# Cost-Effectiveness of Hepatitis B Mass Screening and Management in High-Prevalent Rural China: A Model Study From 2020 to 2049

**DOI:** 10.34172/ijhpm.2021.126

**Published:** 2021-09-07

**Authors:** Xiaolan Xu, Chensi Wu, Lushun Jiang, Chunting Peng, Liya Pan, Xue Zhang, Wei Shen, Lin Chen, Zhuoqi Lou, Kaijin Xu, Lanjuan Li, Yin Dong, Bing Ruan

**Affiliations:** ^1^State Key Laboratory for Diagnosis and Treatment of Infectious Diseases, National Clinical Research Center for Infectious Diseases, Collaborative Innovation Center for Diagnosis and Treatment of Infectious Diseases, The First Affiliated Hospital, College of Medicine, Zhejiang University, Hangzhou, China.; ^2^People’s Hospital Medical Community of Yuhuan County, Taizhou, China.

**Keywords:** Economic Evaluation, Viral Hepatitis, Antiviral Treatment, Adult Vaccination

## Abstract

**Background:** Chronic hepatitis B (CHB) is highly prevalent among adults in rural China and better management of those populations is of vital importance for viral hepatitis elimination. Adult immunization has been the subject of much controversy in previous studies. This study estimates the cost-effectiveness of population-based hepatitis B screening, treatment, and immunization strategy (comprehensive strategy) in rural areas with high prevalence under the national policy of sharp-drop drug prices.

**Methods:** We constructed a Markov model comparing 4 strategies in a 30-year horizon from the healthcare payer perspective: (1) the conventional pattern; (2) screening and treating infected (treatment); (3) screening and immunizing susceptible individuals (immunization); and (4) the comprehensive strategy. Screening intensity ranged from 50% to 100%. Outcomes were measured by costs, quality-adjusted life-years (QALYs), incremental cost-effectiveness ratios (ICERs), and clinical outcomes.

**Results:** The costs for the conventional pattern, treatment strategy, immunization strategy, and comprehensive strategy were US$ 341, 351, 318, and 323, respectively. In addition, effects were 17.45, 17.57, 17.46, and 17.58 QALYs, respectively. The ICER of the comprehensive strategy was US$ 35/QALY gained at 50% screening intensity and 420 US$/QALY gained at 100%. The net monetary benefit increased with increasing screening intensity and declined after 90%, with the highest value of US$40 693. All new infections and 52.5% mortality could be avoided from 2020 to 2049 if all patients were properly treated and all susceptible individuals were immunized. The results were stable within a wide range of parameters.

**Conclusion:** It was cost-effective to implement the mass hepatitis B screening, treatment, and immunization strategy in areas of rural China with high prevalence, and the strategy gained the most net monetary benefit at a screening intensity of 90%. Although it was impractical to fulfill 100% coverage, efforts should be made to obtain more people screened.

## Background

**Key Messages**

** Implications for policy makers**

- Population-based mass hepatitis B virus (HBV) screening, treatment, and immunization strategy for rural residents in high prevalence areas is cost-effective.
- The adult HBV immunization strategy is cost-saving if fewer than 90% of susceptible individuals are vaccinated, and new infection could be reduced by nearly 100% by universal immunization. Free vaccines for adults could be considered.
- Early detection and treatment of HBV infection, and vaccination for susceptible persons is the route we must take to realize HBV elimination by 2030.

** Implications for the public**

This study proves that population-based hepatitis B virus (HBV) screening, treatment, and immunization strategy in rural areas with high prevalence will prevent most new hepatitis B infections if all susceptible individuals are vaccinated. Although it is impractical to make every person get the vaccination, we should endeavor to reach 90% participation in the population. However, there are many HBV patients currently in China. The prevalence is high, especially in the middle-aged and elderly population, because they were not vaccinated. Complications such as liver cirrhosis and hepatocellular carcinoma (HCC) are prone to occur in this population, so mortality decreases slightly (52.5% in this study). Therefore, it is necessary to increase public willingness to participate in HBV screening to increase the detection rate and offer early management and treatment to reduce incidence and mortality. Therapeutic efficiency should be enhanced for patients in progressive stages.

A total of 257 million people were infected with hepatitis B virus (HBV) by 2015 globally, leading to nearly 900 000 HBV-related deaths.^1^ Although the HBV prevalence in children under 5 years of age has dropped to 0.3% in China, there were nearly 100 million HBV infections in 2015.^2,3^ Only 19% of those have been diagnosed, and 10%-11% of those with treatment indications have initiated antiviral treatment, which was far from the World Health Organization (WHO) targets of 90% of HBV-infected diagnosed and 80% on treatment.^3^ A model study predicted that 60 million HBV infections and 680 000 HBV-related deaths would occur in China by 2030 with current practice.^4^

 Long-term treatment could postpone the occurrence of liver cirrhosis or HBV-related hepatocellular carcinoma (HCC) to improve quality of life.^5^ HBV screening and treatment were cost-effective in community-based study in West Africa or with immigrants in North America.^6,7^ Immunization was the most effective way to prevent new infections. The immunization rate in 2006 was less than 10% in susceptible individuals aged 15~59 in China.^8^ Moreover, approximately 40% of those who received immunization at birth had an HBV surface antibody (HBsAb) level below 10 mIU/mL in adolescence, and it was economically beneficial to vaccinate close contacts, individuals with diabetes, immigrants, men who have sex with men, sex workers, incarcerated people, and drug abusers.^9-13^

 HBV prevalence in 2006 in the rural population and unvaccinated individuals aged 1~59 years was 7.3% and 9.4%, respectively.^14^ Since China issued the “National Organization of Drug Centralized Procurement and Use Pilot Program” in 2019, the price of antiviral drugs has decreased sharply, which may greatly mitigate the economic burden of treatment to rural patients. Thus, we carried out an economic evaluation of whether mass screening, treatment, and immunization could be cost-effective in this population with various intervention intensities in the next 30 years.

## Methods

###  Model Structure

 We used TreeAge Pro Healthcare (TreeAge Software, Inc., Williamstown, Massachusetts) to build a Markov decision tree model. The progression after infection was divided into 10 states: immune acquired, immune tolerant, acute hepatitis B, chronic hepatitis B (CHB), inactive HBV surface antigen (HBsAg) carrier, compensated liver cirrhosis, decompensated liver cirrhosis, HBV-related HCC, liver transplantation, and death.^15^ As the disease progressed, transitions occurred between the above disease states. The transition probability was obtained from the published literature (Table 1), and the range was represented by the 95% confidence interval (CI). The maximum and minimum determined in the literature were replaced as the variation range if the 95% CI was absent. If there was only one value available, the variation range was set to ± 25% and constrained between 0 and 1. With 2020 as the entry year, the Markov model ran 30 cycles with a 1-year interval, simulating HBV epidemiology in a fixed cohort. The model was constructed based on the following assumptions and detailed in Supplementary file 1:

1. Except for the mutual transition between CHB and inactive HBsAg carriers, the transition of other disease states was irreversible (Figure S1, Supplementary file 1),^16^ and the transition probability between each state was fixed each year.
2. Individuals with HBsAb titers above 10 mIU/mL would not be infected with HBV.
3. In the conventional pattern, rural patients did not go to a hospital for health examinations on their initiative until exacerbation. While symptoms were obscure in carriers and CHB patients who were discovered in the later stage in most cases, it was assumed that there was no treatment cost in these groups in the conventional pattern. In addition, we hypothesized that comprehensive management of HBV carriers with regular follow-up could reduce their incidence of CHB, liver cirrhosis, and HCC by 50%.
4. The simplified model simulating the natural history of HBV infection and the altered progress after antiviral treatment or immunization was depicted in Figure S2 (Supplementary file 1).

**Table 1:** Main Model Parameters

	**Base Value**	**Deterministic Range**	**PSA Distribution**	**Reference**
General inputs				
Start age of cohort, y	28	28-60	-	-
Discount rate, %	3.00	0.00-6.00	Uniform	^6^
HBsAg positive rate, %	11.10	10.60-11.50	Beta	Supplementary file 2
HBsAb positive rate, %	60.50	59.80-61.20	Beta	Supplementary file 2
Screening intensity, %	50.00	50.00-100.00	-	Assumption
Seroconversion rate after immunization, %	91.50	88.00-95.00	Beta	^17^
Cost inputs				
Annual cost for acute infection, US$	1531.80	-	Gamma	Supplementary file 2
Annual cost for compensated cirrhosis, US$	1252.30	-	Gamma	Supplementary file 2
Annual cost for decompensated cirrhosis, US$	3441.30	-	Gamma	Supplementary file 2
Annual cost for HCC, US$	2267.20	-	Gamma	Supplementary file 2
Annual cost for transplantation (first year, US$)	45 748.00^a^	-	Gamma	^18^
Annual cost for transplantation (subsequent year, US$)	7926.00^a^	-	Gamma	^18^
Annual cost for intervening carriers, US$	75.70	-	Gamma	Supplementary file 2
Annual cost for intervening CHB, US$	205.30	-	Gamma	Supplementary file 2
Immunization cost, US$	50.40	-	Gamma	Supplementary file 2
Screening cost for immunization, US$	10.90	-	-	Supplementary file 2
Screening cost for treatment, US$	29.10	-	Gamma	Supplementary file 2
Annual disease transition rates				
From immunotolerant to immune active, %	3.40	2.55-4.25	Beta	^19^
From CHB to inactive carrier, %	5.70	4.58-6.88	Beta	^6^
Treated CHB to inactive carrier, %	12.60	11.40-13.80	Beta	^12^
From CHB to compensated cirrhosis, %	3.20	2.40-4.00	Beta	^20^
Treated CHB to compensated cirrhosis, %	1.76	1.32-2.20	Beta	^20,21^
From CHB to HCC, %	0.60	0.53-0.72	Beta	^16^
Treated CHB to HCC, %	0.30	0.25-0.35	Beta	^16,21^
From CHB to death, %	0.75	0.00-1.50	Beta	^20^
Treated CHB to death, %	0.45	0.00-0.90	Beta	^20,21^
From inactive carrier to HBsAg clearance, %	1.00	0.00-2.00	Beta	^16^
Treated inactive carrier to HBsAg clearance, %	4.50	2.00-7.00	Beta	^22^
From inactive carrier to CHB, %	4.40	1.60-4.70	Beta	^6,23^
From inactive	0.10	0.08-0.12	Beta	^12^
From inactive carrier to compensated cirrhosis, %	0.10	0.00-0.10	Beta	^16^
From compensated cirrhosis to decompensated cirrhosis, %	4.00	3.00-4.00	Beta	^16^
Treated compensated cirrhosis to decompensated cirrhosis, %	1.80	1.35-1.80	Beta	^16,21^
From cirrhosis to HCC, %	3.70	3.10-4.14	Beta	^16^
Treated cirrhosis to HCC, %	2.00	1.40-4.10	Beta	^24^
From compensated cirrhosis to death, %	3.40	2.90-3.90	Beta	^6,16^
Treated compensated cirrhosis to death, %	1.10	0.70-1.60	Beta	^25^
From decompensated cirrhosis to death, %	26.50	21.40-31.60	Beta	^16^
Treated decompensated cirrhosis to death, %	8.60	7.50-9.70	Beta	^26^
From decompensated cirrhosis to liver transplantation, %	0.65	0.49-0.81	Beta	^27^
From HCC to death, %	14.40	11.60-17.20	Beta	^20^
From HCC to liver transplantation, %	0.65	0.49-0.81	Beta	^27^
Annual mortality rate after liver transplantation, %	7.80	4.90-10.70	Beta	^28^
Annual natural mortality rate, %	0.71^b^	0.53-0.89	Beta	-
From susceptible to infected, %	0.95	0.71-1.19	Beta	^29^
From acute infected to CHB, %	5.00	1.00-10.00	Beta	^30,31^
From acute infected to death, %	0.80	0.30-1.70	Beta	^12^
Utility parameters				
Immune acquired	0.99	0.98-1.00	Beta	^12^
Susceptible	0.99	0.98-1.00	Beta	^12^
Immune tolerant	0.99	0.98-1.00	Beta	^12^
Acute hepatitis B	0.70	0.63-0.77	Beta	^12^
CHB	0.52	0.47-0.57	Beta	^32^
Inactive HBsAg carrier	0.85	0.77-0.93	Beta	^12^
Compensated cirrhosis	0.57	0.52-0.62	Beta	^32^
Decompensated cirrhosis	0.26	0.21-0.31	Beta	^32^
HCC	0.31	0.26-0.36	Beta	^32^
Liver transplantation (first year)	0.41	0.36-0.46	Beta	^32^
Liver transplantation (subsequent year)	0.55	0.50-0.60	Beta	^32^

Abbreviations: PSA, probabilistic sensitivity analysis; HBsAb, HBV surface antibody; HCC, hepatocellular carcinoma; CHB, chronic hepatitis B. ^a^The method used to obtain the data are explained in “Cost estimate” in Supplementary file 2. ^b^Referred from China Population & Employment Statistics Yearbook 2019.

###  Cohort Characteristics

 HBV prevalence in rural coastal areas in Zhejiang province was reported to be high in a community-based survey, ranging from 7.5%~17.0% in different age groups, especially in those aged 20~60, and decreased among people over 60 years old.^33,34^ We selected rural residents aged 28~60 years for modeling. We assumed that this population had never been vaccinated because a free HBV neonatal immunization program was launched in 1992. HBsAg prevalence in this age group was analyzed by serum samples collected during HBV screening in 2019 (Table S1, Supplementary file 2). The costs, probabilities, and outcomes involved in this model were set to be independent of age and gender.

###  Comparators

 Based on current HBV screening, we compared the treatment and immunization strategy (comprehensive) with: (1) treatment and management for HBsAg positive individuals (treatment); (2) immunization for susceptible individuals (HBsAg and HBsAb negative) (immunization); and (3) the conventional pattern. No intervention was performed in the conventional pattern, and the population was not screened for HBV infection, so individuals living with HBV may not be found in a timely manner. They do not usually go to the hospital until severe symptoms develop, often caused by liver cirrhosis or HCC. This may cause a delay in the timing of treatment; additionally, hidden patients are not conducive to the control of new infections. We screened HBV serological markers in the immunization strategy and HBV DNA quantification, liver function, and liver ultrasonography additionally in the other two strategies. Immunization was carried out in the first year of the simulation cohort.

 We have carried out HBV screening in our demonstration areas since 2009, and more than 50% of rural residents participated in the screening (more details are provided in part 3 of Supplementary file 2). Therefore, we hypothesized that the basic screening intensity was 50%. Given the actual situation, the higher the screening intensity, the greater the benefits gained, but the intervention cost might increase nonlinearly. Cost increased by 50% with each additional 10% screened. Thus, different screening intensities were simulated and the cost-effectiveness could be calculated to explore the best screening strategy suitable for local conditions.

 Referring to the Guideline of Prevention and Treatment for Chronic Hepatitis B in China, we formulated a treatment and follow-up scheme for each disease state: (1) all HBV infected states except for immune tolerant, acute hepatitis B, and inactive HBsAg carriers should take antiviral drugs; (2) immune tolerant and inactive HBsAg carriers were followed up every six months for routine blood tests, biochemical tests, HBV serological markers, alpha fetoprotein, liver ultrasonography and elastography; (3) patients treated with nucleos(t)ide analogs were followed up every three months for routine blood tests, biochemical tests, HBV DNA quantification, and HBV serological markers; and (4) patients with cirrhosis should be inspected for alpha fetoprotein, liver ultrasonography and elastography every three months, and patients without cirrhosis were inspected for the above items every six months.

###  Estimating Costs

 The costs were divided into four components: screening costs, immunization costs, costs related to health states, and treatment costs. The cost of the screening items referred to the pricing of local secondary hospitals (Table S2, Supplementary file 2). The immunization costs included vaccine fees and inoculation service fees. For costs related to health states, we only calculated direct medical expenses (including registration fees, drug fees, diagnosis and treatment fees, hospitalization fees, examination fees, etc) in the model (more details for costs are provided in Supplementary file 2). We calculated the outpatient expenses under each disease state separately according to the Guideline of Prevention and Treatment for Chronic Hepatitis B in China, and the hospitalization expenses were obtained from previous literature and the electronic medical record system of the People’s Hospital of Yuhuan county (Table S3, Supplementary file 2). For treatment costs, the cost of standard follow-up and treatment for the screened inactive carriers and CHB patients was considered. We calculated the total cost from the healthcare payer perspective. Costs and health benefits were discounted at a discount rate of 3% per year.^6^ All costs are presented in US$, with an exchange rate of US$1 = 6.8985 China Yuan in 2019.

###  Outcome Measurement and Cost-Effectiveness

 Outcomes were presented by (1) average costs; (2) average life-years; (3) average quality-adjusted life-years (QALYs); and (4) clinical outcomes including new HBV infections, compensated or decompensated cirrhosis, HBV-related HCC, liver transplantation and mortality. Utilities for hepatitis-related health states elicited from mainland China patients in the study of Levy et al were adopted.^32^ The range of health utilities varied by 0.05 if a 95% CI was lacking. The incremental cost-effectiveness ratio (ICER) was used to compare the economic feasibility of each intervention, which meant the cost needed to gain an additional QALY. An intervention was supposed to be cost-effective with an ICER lower than the willingness-to-pay (WTP) threshold. The WHO recommends that an ICER less than three times the annual gross domestic product (GDP) per capita is cost-effective, while less than one times the GDP per capita is extremely cost-effective.^35^ The GDP per capita in 2019 was US$10 276 in China.^36^ Given the rural population selected in our study, we adopted a more rigorous criterion, the disposable income of rural residents per capita in 2019 (US$2322), as the appointed WTP.

###  Sensitivity Analysis

 The parameters involved in the model varied within wide ranges. We performed a one-way sensitivity analysis of the screening intensity to determine the most appropriate intensity. Then, we performed a one-way sensitivity analysis for all parameters at the preferred intensity and presented the results through a tornado diagram. The variation range of the costs was set to ± 50%. A two-way sensitivity analysis was conducted to determine the preferred strategy when the screening costs in the comprehensive strategy and screening intensity were altered synchronously. Multivariate probabilistic sensitivity analysis (PSA) reflected the probability that interventions were cost-effective within the WTP threshold when all parameters changed simultaneously. Distributions for parameters were specified by gamma distributions for costs (range of ± 50%), beta distributions for probabilities, epidemiological parameters, and utilities, and uniform distributions for discount rate and screening intensity. The beta distributions were derived by the calculation method with the mean and standard deviation. The mean was the base value in Table 1, and the standard deviation was half of the difference of the upper and lower limits of the deterministic range. The PSA results were presented in the form of a cost-effectiveness acceptability curve.

## Results

###  Costs, QALYs, and Cost-Effectiveness at Baseline Intensity

 The model has been validated to be consistent with real-world epidemic data (Table S4, Supplementary file 1). In the conventional pattern, the average cost per person was US$341 and obtained 18.08 life-years and 17.45 QALYs, respectively. In the treatment strategy, the average cost was US$351 and obtained 18.12 life-years and 17.57 QALYs, respectively. In the immunization strategy, the average cost was US$318 and obtained 18.08 life-years and 17.46 QALYs, respectively. In the comprehensive strategy, the average cost was US$323 and obtained 18.12 life-years and 17.58 QALYs (Table 2). The immunization strategy cost the least, and the ICER of the comprehensive strategy was US$35/QALY gained compared to the comparative strategy. The conventional pattern and the treatment strategy were dominated by higher costs but lower benefits compared to the comprehensive strategy (Figure S3, Supplementary file 3).

**Table 2:** Summary of Average Outcomes and ICERs at a Screening Intensity of 50%

**Strategy**	**Average Per Person**			**Incremental**			**ICERs, US$/QALY**
	**Cost, US$**	**QALY**	**Life-Year**	**Cost, US$**	**QALY**	**Life-Year**	
Immunization	318	17.46	18.08	-	-	-	-
Comprehensive	323	17.58	18.12	4.5	0.13	0.04	35
Conventional pattern	341	17.45	18.08	18.3	-0.14	-0.05	Dominated
Treatment	351	17.57	18.12	28.7	-0.01	0	Dominated

Abbreviations: ICER, incremental cost-effectiveness ratio; QALY, quality-adjusted life-year. The willingness-to-pay threshold is US$2322 per QALY gained.

###  Cost-Effectiveness Analysis for Various Screening Intensities 

**Figure 1:**
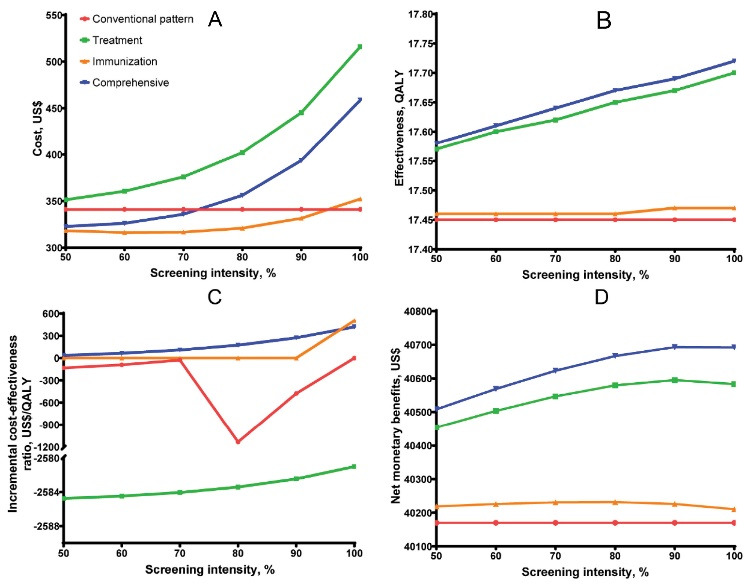
Cost-Effectiveness Analysis Results for Different Screening Intensities. Abbreviation: QALY, quality-adjusted life-year.

 The costs, QALYs, and ICERs increased with the treatment and comprehensive strategy as the screening intensity increased (Figure 1A, 1B, 1C). However, the cost of the immunization strategy tended to decrease first and then increase, but it kept the most cost savings among the four strategies if the intensity was lower than 90% (Figure 1A). The conventional pattern and treatment strategy were always inferior to the other two strategies as the screening intensity changed (Figure 1C). Except for the conventional pattern, the net monetary benefits of the other three strategies all increased with increasing screening intensity and decreased after 90% (Figure 1D). The comprehensive strategy gained the most net monetary benefits, with the highest of US$40 693 at the screening intensity of 90%. Compared with the lowest cost strategy, the ICERs of the comprehensive strategy were 65, 108, 174, 272, and 420 US$/QALY gained when screening 60%, 70%, 80%, 90%, and 100% of the population, respectively, which were still lower than the WTP (Table S5, Supplementary file 3).

###  Reduction of Clinical Outcomes

**Figure 2:**
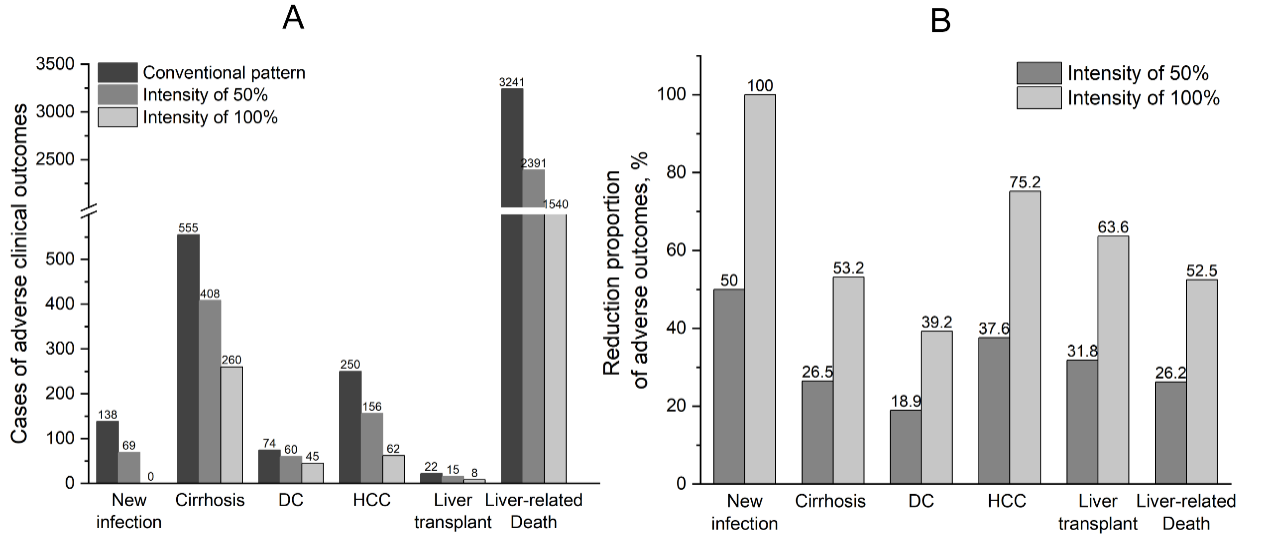
Clinical Outcomes of the Conventional Pattern and Comprehensive Strategy With Different Screening Intensities. (A) Cases of clinical outcomes for the conventional pattern and comprehensive strategy with an intensity of 50% or 100%; (B) The reduction percentage of clinical outcomes. Abbreviations: DC, decompensated cirrhosis; HCC, hepatocellular carcinoma.

 We established a simulating cohort of 10 000 HBV patients and 10 000 susceptible individuals to compare the clinical outcomes of the comprehensive intervention and conventional pattern. New infection, compensated cirrhosis, decompensated cirrhosis, HBV-related HCC, liver transplantation, and liver-related mortality would decrease by 50%, 26.5%, 18.9%, 37.6%, 31.8%, and 26.2%, respectively, in 2049 if the screening intensity was 50%. The above decreased by 100%, 53.2%, 39.2%, 75.2%, 63.6%, and 52.5%, respectively, while the screening intensity reached 100% (Figure 2). Fourteen liver transplantations could be avoided for every 10 000 HBV-infected individuals if population-wide comprehensive intervention was performed.

###  Sensitivity Analysis

**Figure 3:**
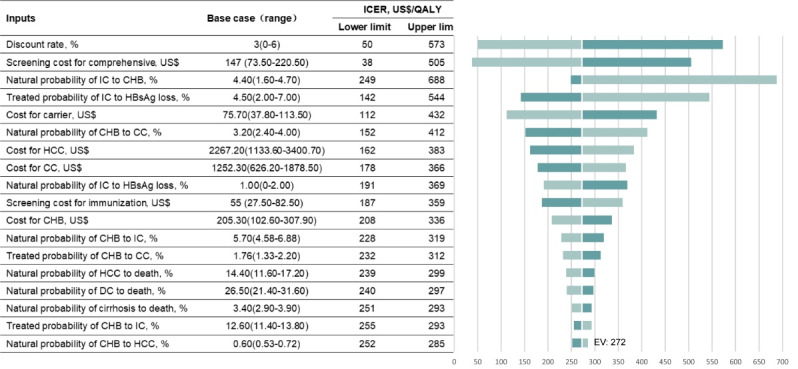
One-Way Sensitivity Analysis of ICERs at a Screening Intensity of 90% (the Comprehensive Strategy vs. the Immunization Strategy). The willingness-to-pay threshold is US$2,322 per QALY gained. The dark green portion of each bar represents the high range of the parameter, and the light green portion of the bar represents the low range of the parameter. When light green is on the left and dark green is on the right, the ICER increases as the parameter value increases. When dark green is on the left, the ICER decreases as the parameter value increases. The white dashed line indicates the ICER for baseline parameters, which is 272 US$/QALY gained in the figure. Abbreviations: ICER, incremental cost-effectiveness ratios; IC, inactive HBsAg carrier; CHB, chronic hepatitis B; CC, compensated cirrhosis; HCC, hepatocellular carcinoma; DC, decompensated cirrhosis.

Figure 1 showed that the comprehensive strategy at a screening intensity of 90% gained the most net monetary benefit. For this intensity, the ICER was 272US$/QALY gained for baseline parameters compared to the immunization strategy. Therefore, we displayed the 18 parameters most influential on ICERs at the screening intensity of 90% in the tornado diagram in Figure 3. The discount rate, screening cost for the comprehensive strategy, natural transition probability from inactive carrier to CHB, and annual HBsAg clearance rate after antiviral treatment were the four most influential parameters in the model in which the upper limit was more than twice the baseline value. The maximum ICER of the comprehensive strategy was 688US$/QALY gained if the natural transition probability from inactive carrier to CHB was 1.6%. Thus, one-way sensitivity analysis showed that the comprehensive strategy was always cost-effective over the wide variety of all parameters.

 The results from the two-way sensitivity analysis revealed that the comprehensive strategy always remained cost-effective, although the screening costs and intensity varied in the deterministic range (Figure S4, Supplementary file 3).

 We then performed 10 000 Monte Carlo Simulations and the PSA results were shown in Figure S5 and Figure S6 (see Supplementary file 3). The probability that the comprehensive strategy would be cost-effective was 98.4% at a WTP threshold of 2322US$/QALY when all parameters were sampled randomly.

## Discussion

 Our study suggested that comprehensive intervention, including mass screening, treatment, and immunization, was cost-effective after 30 years of dynamic changes under the current HBV background in rural areas with high prevalence. However, Hutton et al indicated that based on screening and treatment, immunization for close contacts of HBsAg positive patients was more cost-effective than universal immunization (US$39 903/QALY gained).^13^ Rossi et al reported that the cost of adult immunization was too high and was inferior to the treatment alone strategy.^7^ In regard to the high-risk population, the comprehensive strategy often turned to be cost-effective. For example, Chahal et al found that the screening, treatment, and immunization strategy was cost-effective among six high-risk populations in the United States (US$3203/QALY gained).^12^ Immunization provided for all HBsAg-negative wives of HBsAg-positive husbands could improve the efficiency of preventing mother-to-child transmission and was cost-effective in China.^37^ Tatar et al compared the cost-effectiveness of universal and targeted hepatitis C virus screening and found that screening and treatment may be cost-effective for people who inject drugs but not for the general population.^38^ However, sensitivity analysis showed that if the treatment cost decreased to $13 200 per patient and the infection rate increased to 10%, universal screening would be cost-effective. Such differences may be caused by the difference in cost parameters and HBV prevalence in different countries and regions. The centralized drug procedure and use policy in the recent year has caused a sharp reduction in treatment expense for HBV patients, making the comprehensive strategy more affordable and feasible for rural residents. In other words, reducing the medical expense for patients was a stepping stone to eliminating viral hepatitis, which provided a framework for formulating HBV-related health policies in other countries and regions.

 Our study set a basis screening intensity of 50%, that is, half of the population was active in participating in the screening. As screening intensity increased, more labor and financial resources were required to convince more people to participate. We quantified this characteristic through exponential screening costs and thereby analyzed the cost-effectiveness of various screening intensities, which was not described in other studies. Identifying hidden HBV patients and immunizing susceptible persons are essential methods to inhibit the spread of HBV. Our study indicated that the comprehensive strategy remained cost-effective compared to the other three strategies in high-prevalence and low-income areas. In addition, it had the most net monetary benefit at a screening intensity of 90% (Figure 1D). Sensitivity analysis revealed that the results remain robust among a wide variety of parameters. Considering that we used the disposable income of rural residents per capita as WTP, our results could be applied in more developed regions.

 According to our results, new HBV infections could be eliminated if all susceptible individuals were immunized, but this is impractical. Efforts should be made to achieve immunization coverage over 90%. However, liver-related mortality could only be reduced by 52.5% with 30-year normative treatment in 2049. Thus, the efficacy of antiviral drugs must be enhanced to reduce mortality. Recently, scientists were exploring a functional cure, and nucleos(t)ide analogs combined with interferons or sequential therapy were superior to monotherapy in HBsAg loss in a subset of patients.^39^ Besides, other therapeutic strategies, such as the artificial liver support system and liver transplantation, should be employed in severe cases.

 We calculated the cost from the perspective of the healthcare payer. Our study carried out an economic evaluation based on the sharp reduction in antiviral drug price and took the screening intensity into account. Different from previous studies, the cost of outpatients was simulated following the treatment and follow-up recommended in the Guideline of Prevention and Treatment for Chronic Hepatitis B in China, therefore reflecting the cost-effectiveness of the comprehensive strategy under standard management regimens. Moreover, we simulated a dynamic transition of HBV in the stable cohort, but population mobility was not considered.

 Our study has several limitations. First, we used average costs, utilities, and transition probabilities without considering the effects of age, gender, or comorbidities. Second, the Markov model did not distinguish different HBV genotypes or distinguish HBV e antigen-positive and HBV e antigen-negative CHB. Third, our model ignored consolidation treatment and regular follow-up within one year after the withdrawal of antiviral drugs recommended by the guideline (monthly biochemical routine, HBV serological markers, and HBV DNA quantification in the first three months and once every three months afterward). Finally, we did not consider any adjunctive drugs other than antivirals in the outpatient cost, nor the adverse effects or resistance of antiviral therapy, and the hospitalization cost was obtained from one medical center, but the sensitivity analysis compensated for this to some extent.

 Parameters in this model were obtained from published literature and field surveys. The cost-effectiveness results remained robust after we changed the epidemiological parameters and cost parameters, so they can be generalized to other areas of China. Before the intervention strategies were put into practice, we need to consider the affordability for the local government and how to enhance the willingness of residents to participate. Previous practice has shown that combining the HBV screening program with other government policies, such as health examinations, is an approach to increase screening intensity. In addition, with the wide coverage of contracted services by general practitioners in China, we are trying to implement a new HBV management pattern in Zhejiang demonstrated areas, that is, a single-disease contract for HBV to provide specific health management for patients.

## Conclusion

 Generally, the strategy of mass HBV screening, immunization for susceptible persons, and treatment for infected patients is of great significance to achieving the goal of eliminating hepatitis B by 2030 and should be put into practice in rural areas with high prevalence. Centralized procurement in China has greatly reduced the price of drugs, and this experience could be used in other countries and regions to reduce the disease burden in resource-limited areas and make universal intervention cost-effective.

## Ethical issues

 The study has been approved by the Human Research Ethics Committee of the First Affiliated Hospital, College of Medicine, Zhejiang University with informed consent (No. 2017-376).

## Competing interests

 Authors declare that they have no competing interests.

## Authors’ contributions

 Conception and design: BR. Acquisition of data: LJ, CP, and LP. Analysis and interpretation of data: XX and CW. Drafting of the manuscript: XX. Critical revision of the manuscript for important intellectual content: XZ and BR. Obtaining funding: BR. Administrative, technical, or material support: WS, LC, and ZL. Supervision: LL, KX, and YD.

## Funding

 The work was supported by The National Science and Technology Major Project of China (Grant No. 2017ZX10105001) and National Human Genetic Resources Sharing Service Platform (Grant No. 2005DKA21300).

## Supplementary files


Supplementary file 1. Markov Model Information.

Supplementary file 2. Epidemiological and Cost Parameters.

Supplementary file 3 contains Table S5 and Figures S3-S6.
