# Supplementary material for: Cost-Effectiveness of Hepatitis B Mass Screening and Management in High-Prevalent Rural China: A Model Study From 2020 to 2049
Source: Int J Health Policy Manag. 2021 Sep 7;11(10):2115–23. doi: 10.34172/ijhpm.2021.126 (PMC9808295; doi:10.34172/ijhpm.2021.126)
Supplement: Supplementary file 1 — Markov Model Information. [file ijhpm-11-2115-s001.pdf]

Article title: Cost-Effectiveness of Hepatitis B Mass Screening and Management in High-Prevalent Rural China: A Model Study From 2020 to 2049

Journal name: International Journal of Health Policy and Management (IJHPM)

Authors' information: Xiaolan Xu[1]¶, Chensi Wu[1]¶, Lushun Jiang[1], Chunting Peng[1], Liya Pan[1], Xue Zhang[1], Wei Shen[1], Lin Chen[1], Zhuoqi Lou[1], Kaijin Xu[1], Lanjuan Li[1], Yin Dong[2]*, Bing Ruan[1]*

[1]State Key Laboratory for Diagnosis and Treatment of Infectious Diseases, National Clinical Research Center for Infectious Diseases, Collaborative Innovation Center for Diagnosis and Treatment of Infectious Diseases, The First Affiliated Hospital, College of Medicine, Zhejiang University, Hangzhou, China.

[2]People's Hospital Medical Community of Yuhuan County, Taizhou, China.

¶Both authors contributed equally to this paper.

(*Corresponding author: Yin Dong; Email: 9597082@qq.com &

Bing Ruan; Email: ruanbing@zju.edu.cn)

Supplementary file 1. Markov Model Information

1. Model assumptions

Modeling natural history:

(1) Except for the mutual transition between CHB and inactive HBsAg carriers, the transition of other disease states was irreversible (Figure S1),[1] and the transition probability between each state was fixed each year.

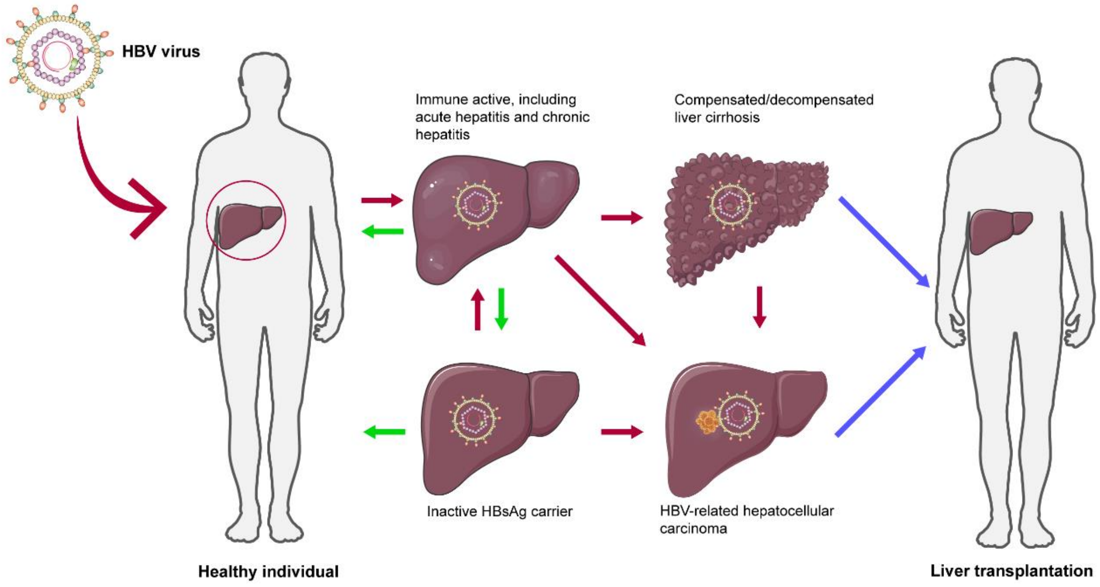

Figure S1. Natural history and disease progression for hepatitis B infected adults. The green arrows represented the favorable prognosis, the red arrows represented the adverse prognosis, and the blue ones represented liver transplantation. All states could lead to an absorbing state of death in Markov model. The immune tolerant state was absent in adult-acquired HBV infection. Studies have found inactive carriers even had a risk below 0.1 per 100-person years of cirrhosis development, which was not shown in the figure.

(2) Individuals with HBsAb titer above 10 mIU/mL would not be infected with HBV.

(3) Adult-acquired HBV infection entered the acute hepatitis B state and transitioned into either CHB or HBsAg clearance state. Asymptomatic and symptomatic acute hepatitis, fulminant hepatitis, and death from fulminant hepatitis were modeled in this state. Liver transplantation caused by fulminant hepatitis was not considered in this state because of the low incidence in this population.

(4) Perinatal-acquired HBV infection entered either CHB, immune tolerant, or inactive carrier state.

(5) Cirrhosis or HCC developed from patients who have achieved HBsAg clearance was not modeled.

(6) HBV reoccurrence in patients who suffered liver transplantation was not modeled.

<u>Modeling screening:</u>

(1) The baseline screening intensity was 50%, representing half of the population were willing to participate in screening currently.

(2) If more people were mobilized to participate in screening, more resources must be consumed, which was reflected by the higher cost of screening. We assumed screening cost increased by 50% with each additional 10% population screened.

(3) We didn't consider the probability of false negatives or false positives with screening tests.

<u>Modeling treatment:</u>

(1) In the conventional pattern, rural patients don't go to a hospital for health examinations on their initiative until exacerbation. While symptoms were obscure in carriers and CHB patients which were discovered in the later stage in most cases, so it was assumed that there was no treatment cost in these groups in the conventional pattern.

(2) In the treatment or comprehensive strategy, carriers and CHB patients were discovered and managed according to the guideline. There would be outpatient costs from these patients, but no hospitalization.

(3) We hypothesized that comprehensive management of HBV carriers with regular follow-up could reduce their incidence of CHB, liver cirrhosis, and HCC by 50%.

(4) Antiviral treatment was initiated in CHB patients, not in immune tolerant individuals or carriers.

(5) Drug resistance was not considered in the model.

(6) Inactive carriers and CHB patients were assumed no hospitalization costs because these patients were either unaware of their infection in the conventional pattern or well managed in the treatment or comprehensive strategy. While acutely infected persons, cirrhosis, or HCC patients had hospitalization costs annually if patients kept in the state.

<u>Modeling immunization:</u>

(1) By default, those who were willing to be vaccinated would accomplish all three doses, and those with HBsAb titer > 10 mIU/mL would not be infected.

2. The construction of Markov model

According to the HBV serological markers, HBV DNA quantification, alpha fetoprotein, alanine transaminase or aspartate aminotransferase abnormality, liver fibrosis degree, and liver occupation or not, the progression after HBV infected was divided into 10 states and the simplified Markov model was shown in Figure S2. The disease distribution of the initial state of the cohort entered the model was assumed with similar data in former studies. Ratios for immune tolerant, chronic hepatitis B (CHB), inactive HBsAg carriers, compensated cirrhosis, and decompensated cirrhosis were 1.5%, 5%, 90.5%, 2.5%, and 0.5%, respectively.[2] And the transition probability between above states were referred from published researches. We used the following formula to calculate the annual transition rate when it was unavailable.[3]

$$r=-\frac{1}{t}\ln(1-p),$$

$$Pr(annual)=1-exp(-r)$$

(r represents annual transition rate, t represents the time of follow-up, p represents the cumulative incidence obtained from long-term follow-up in the literature, and Pr represents annual transition probability)

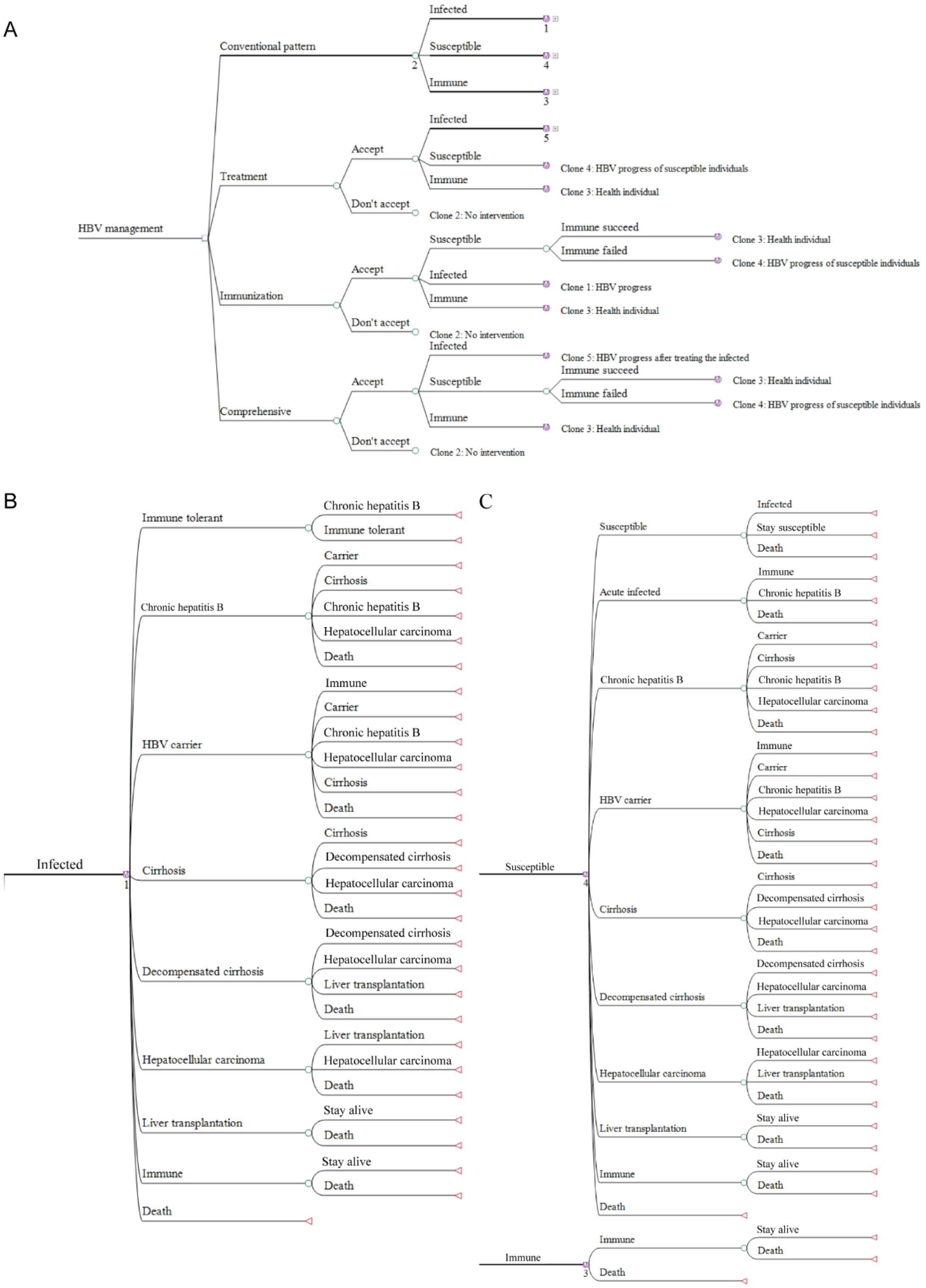

Figure S2. Simplified diagram of the Markov decision tree model. (A) This model illustrated the progress of HBV patients and susceptible individuals under different intervention strategies. After the population entered the model, they would receive conventional pattern, screening for infected and treatment, screening for susceptible

and immunization, or screening for both with treatment or immunization. Patients could choose to accept or not, which reflected the screening intensity. Infected represented people with HBsAg positive, susceptible represented people with HBsAg and HBsAb negative, and immune refers to people who have acquired HBV immunity with positive HBsAb. (B) the expanded subtree of clone 1 and 5. (C) The expanded subtree of clone 3 and 4. HBV, hepatitis B virus; HBsAg, HBV surface antigen; HBsAb, HBV surface antibody.

3. Model calibration and validation

We compared the HBV infection progress in natural history and after antiviral treatment with published epidemiological surveys or research articles. The distribution for CHB patients at the initial state of the infection cohort was set to 1 to simulate the incidence of liver cirrhosis and HCC in the population after 5 and 48 years, respectively. Similarly, we set the distribution for inactive HBsAg carriers to 1 and calculate the cumulative incidence of HBsAg loss and hepatitis recurrence. The distribution for compensated cirrhosis was set to 1 to calculate the cumulative incidence of decompensation and HCC. Besides, cumulative mortalities in CHB, cirrhosis, and HCC were also simulated. To be mentioned, we assumed that the annual mortality rate of HCC kept the same in the 4 comparators for patients with HCC would seek for help from doctors initiatively in the conventional pattern, and treatment was unnecessary for inactive carriers. Hence, we didn’t simulate the incidence after antiviral treatment in these circumstances. We conducted the Markov cohort analysis of a fixed cohort of 1000 people with the following results in Table S4. It was consistent with the results of published cohort studies or observational studies, indicating that the Markov model we established was in line with real-world data and feasible for the subsequent cost-effectiveness analysis.

Table S4. Validation results for HBV progress in natural history and after antiviral treatment

**Primary state**	**Target state**	**Time horizon**	**Natural history**		**Antiviral treatment**	
			**Cumulative incidence**	**Reference**	**Cumulative incidence**	**Reference**
Inactive carrier	Chronic hepatitis B	5-year	19.4%	9.7-25.2%[4,5]	-	-
	HBsAg clearance	10-year	7.7%	8.1%[6]	-	-
Chronic hepatitis B	Compensated cirrhosis	5-year	13.1%	8-17%[1]	6.6%	2.9-8.1%[7*]
		48-year	42.7%	41.5%[8]	-	-
	Hepatocellular carcinoma	5-year	2.8%	1-3%[1]	1.2%	1-1.2%[9,10]
		48-year	22.5%	21.7%[8]	-	
	Death	5-year	3.1%	<4%[11]	1.7%	0.5-1.3%[12]
Compensated cirrhosis	Decompensated cirrhosis	5-year	16.1%	15-20%[13]	3.5%(2-year)	3.4-3.6%[14]
	Hepatocellular carcinoma	5-year	14.8%	10-17%[1]	9%	4-9%[9,10]
	Death	5-year	13.6%	14-15%[1]	4.9%	3.6-6.4%[12]
Decompensated cirrhosis	Death	5-year	72.4%	70-85%[1]	34.3%	39.9%[15]
Hepatocellular carcinoma	Death	10-year	76.9%	69-99.1%[16 ※]	-	-

[*]Chronic hepatitis B patients with antiviral therapy have significant risk reductions in cirrhosis compared to no therapy in random clinical trials (risk ratio=0.55; 95% confidence interval: 0.38–0.78), according to which we calculate 5-year cumulative incidence rate. [※]Data referred from survival analysis data after relevant treatment.

References:

1. Fattovich G, Bortolotti F, Donato F. Natural history of chronic hepatitis B: special emphasis on disease progression and prognostic factors. *J Hepatol*. 2008;48(2):335-352. doi: 10.1016/j.jhep.2007.11.011

2. Nayagam S, Conteh L, Sicuri E, et al. Cost-effectiveness of community-based screening and treatment for chronic hepatitis B in The Gambia: an economic modelling analysis. *The Lancet Global health*. 2016;4(8):e568-578. doi: 10.1016/s2214-109x(16)30101-2

3. Fleurence RL, Hollenbeak CS. Rates and probabilities in economic modelling: transformation, translation and appropriate application. *Pharmacoeconomics*. 2007;25(1):3-6. doi: 10.2165/00019053-200725010-00002

4. Chu CM, Liaw YF. Incidence and risk factors of progression to cirrhosis in inactive carriers of hepatitis B virus. *Am J Gastroenterol*. 2009;104(7):1693-1699. doi: 10.1038/ajg.2009.187

5. Tseng TC, Liu CJ, Chen CL, et al. Serum hepatitis B virus-DNA levels correlate with long-term adverse outcomes in spontaneous hepatitis B e antigen seroconverters. *J Infect Dis*. 2012;205(1):54-63. doi: 10.1093/infdis/jir687

6. Chu CM, Liaw YF. HBsAg seroclearance in asymptomatic carriers of high endemic areas: appreciably high rates during a long-term follow-up. *Hepatology*. 2007;45(5):1187-1192. doi: 10.1002/hep.21612

7. Terrault NA, Bzowej NH, Chang KM, Hwang JP, Jonas MM, Murad MH. AASLD guidelines for treatment of chronic hepatitis B. *Hepatology*. 2016;63(1):261-283. doi: 10.1002/hep.28156

8. Chen CJ, Yang HI. Natural history of chronic hepatitis B REVEALed. *J Gastroenterol Hepatol*. 2011;26(4):628-638. doi: 10.1111/j.1440-1746.2011.06695.x

9. Papatheodoridis GV, Manolakopoulos S, Touloumi G, et al. Hepatocellular carcinoma risk in HBeAg-negative chronic hepatitis B patients with or without cirrhosis treated with entecavir: HepNet.Greece cohort. *J Viral Hepatitis*.

2015;22(2):120-127. doi: 10.1111/jvh.12283

10. Buti M, Fung S, Gane E, et al. Long-term clinical outcomes in cirrhotic chronic hepatitis B patients treated with tenofovir disoproxil fumarate for up to 5 years. *Hepatol Int*. 2015;9(2):243-250. doi: 10.1007/s12072-015-9614-4

11. Lin X, Robinson NJ, Thursz M, et al. Chronic hepatitis B virus infection in the Asia-Pacific region and Africa: review of disease progression. *J Gastroenterol Hepatol*. 2005;20(6):833-843. doi: 10.1111/j.1440-1746.2005.03813.x

12. Wong GL, Chan HL, Mak CW, et al. Entecavir treatment reduces hepatic events and deaths in chronic hepatitis B patients with liver cirrhosis. *Hepatology*. 2013;58(5):1537-1547. doi: 10.1002/hep.26301

13. Sundaram V, Kowdley K. Management of chronic hepatitis B infection. *BMJ (Clinical research ed)*. 2015;351:h4263. doi: 10.1136/bmj.h4263

14. Wu X, Shi Y, Zhou J, et al. Combination of entecavir with thymosin alpha-1 in HBV-related compensated cirrhosis: a prospective multicenter randomized open-label study. *Expert Opin Biol Ther*. 2018;18(sup1):61-69. doi: 10.1080/14712598.2018.1451511

15. Jang JW, Choi JY, Kim YS, et al. Effects of Virologic Response to Treatment on Short- and Long-term Outcomes of Patients With Chronic Hepatitis B Virus Infection and Decompensated Cirrhosis. *Clin Gastroenterol Hepatol*. 2018;16(12):1954-1963.e1953. doi: 10.1016/j.cgh.2018.04.063

16. Gluer AM, Cocco N, Laurence JM, et al. Systematic review of actual 10-year survival following resection for hepatocellular carcinoma. *HPB (Oxford)*. 2012;14(5):285-290. doi: 10.1111/j.1477-2574.2012.00446.x
