# Supplementary material for: Cost-Effectiveness of Hepatitis B Mass Screening and Management in High-Prevalent Rural China: A Model Study From 2020 to 2049
Source: Int J Health Policy Manag. 2021 Sep 7;11(10):2115–23. doi: 10.34172/ijhpm.2021.126 (PMC9808295; doi:10.34172/ijhpm.2021.126)
Supplement: Supplementary file 2 — Epidemiological and Cost Parameters. [file ijhpm-11-2115-s002.pdf]

Article title: Cost-Effectiveness of Hepatitis B Mass Screening and Management in High-Prevalent Rural China: A Model Study From 2020 to 2049

Journal name: International Journal of Health Policy and Management (IJHPM)

Authors' information: Xiaolan Xu[1]¶, Chensi Wu[1]¶, Lushun Jiang[1], Chunting Peng[1], Liya Pan[1], Xue Zhang[1], Wei Shen[1], Lin Chen[1], Zhuoqi Lou[1], Kaijin Xu[1], Lanjuan Li[1], Yin Dong[2]*, Bing Ruan[1]*

[1]State Key Laboratory for Diagnosis and Treatment of Infectious Diseases, National Clinical Research Center for Infectious Diseases, Collaborative Innovation Center for Diagnosis and Treatment of Infectious Diseases, The First Affiliated Hospital, College of Medicine, Zhejiang University, Hangzhou, China.

[2]People's Hospital Medical Community of Yuhuan County, Taizhou, China.

¶Both authors contributed equally to this paper.

(*Corresponding author: Yin Dong; Email: 9597082@qq.com &

Bing Ruan; Email: ruanbing@zju.edu.cn)

Supplementary file 2. Epidemiological And Cost Parameters

1. Epidemiological characteristics

China launched the Prevention and Control of Major Infectious Diseases Project in 2009 and established demonstration areas in 7 provinces and cities across the country. Local rural residents were screened for hepatitis B virus (HBV) infection every two year in demonstration areas in Zhejiang Province. The screening program included HBV surface antigen (HBsAg) and antibody (HBsAb). We collected 5ml of venous blood from each participant, and their demographic information including name, sex, age, identity card number, and address was recorded. Samples were stored at a temperature of 0~4 degree centigrade and delivered to KingMed Diagnostics Co., Ltd. (Hangzhou, China) for serologic testing. HBsAg and HBsAb were inspected by commercially available enzyme immunoassay kits (Wantai Biological Pharmacy Co., Ltd., Beijing, China) and samples with HBsAg positive were verified by quantitative kits (Abbott Laboratories, Chicago, IL, USA). Excel 2016 (Microsoft Corporation, Redmond, Washington, USA) and SPSS Statistics 20.0 software (International

Business Machines Corporation, Armonk, New York, USA) were used to analyze the data. We calculated the prevalence of HBsAg and HBsAb with 95% confidence interval (CI) in residents aged 28~60 years. We collected a total of 67,764 serum samples with intact demographic information from rural residents who participated HBV screening in 2019. Finally, 20,974 samples of participants aged 28~60 years were included in the analysis. The HBsAg positive rate was 11.1% (95%CI: 10.6-11.5%) and the HBsAb positive rate was 60.5% (95%CI: 59.8-61.2%) (Table S1).

Table S1. Prevalence of HBV surface antigen and antibody in rural residents aged 28~60 years

Serum marker	Total (N)	Positive individuals (N)	Positive rate, %	95% CI, %
HBsAg	20974	2319	11.1	10.6-11.5
HBsAb	20974	12690	60.5	59.8-61.2

CI, confidence interval; HBsAg, HBV surface antigen; HBsAb, HBV surface antibody

2. Costs estimate

2.1 Screening cost

The inspection items for different intervention strategies were different. We calculated the cost according to the pricing of the laboratory inspections in secondary hospitals in Zhejiang Province (Table S2). For the treatment strategy, the HBV serological markers, HBV DNA quantification, liver function, and liver ultrasonography were necessary with US$ 29.1 in total. While the item of HBV serological markers was enough for the immunization strategy with the cost of US$ 10.9. We set a basic screening intensity of 50%. As the screening intensity varied between 50% and 100%, the screening cost raised 50% than the former each a 10% increase of the inspected population.

Table S2. Details for screening and follow-up items, immunization, and treatment costs

Item	Unit price (US$)	Item	Unit price (US$)
HBV serological markers	10.9	Ultrasonic elastography	7.2
HBV DNA quantification	11.6	Alpha fetoprotein test	3.6
Liver function	4.5	Registration fee	1.7
Blood Routine	2.2	Antiviral drug (/month)	1.6
Liver ultrasonography	2.2	Vaccine (/dose)	12.8
Biochemical routine	10.0	Inoculation fee (/dose)	4.1

2.2 Immunization cost

For people with HBsAg and HBsAb negative, we provide 3 doses of vaccination according to the 0,1, and 6-month immunization procedure. The vaccine used in our model was a Recombinant Hepatitis B Vaccine (CHO Cell) produced by North China Pharmaceutical Jintan Biotechnology Co., Ltd. (Shijiazhuang, China), with a specification of 20 μg/1.0 ml/piece. The price was US$ 12.8 per dose and the inoculation service fee was US$ 4.1 per dose prescribed by the Zhejiang Price Bureau.

2.3 Costs related to health states

Hospitalization cost and outpatient cost were discussed in different health states. Direct non-medical costs and indirect costs were relatively low in the research population, so we didn't take these expenses into consideration in our study.

2.3.1 Hospitalization cost

We searched for patients aged 28-60 years who were hospitalized with HBV-related diseases from February 2012 to October 2019 in the People's Hospital of Yuhuan county. Acute hepatitis B (AHB), compensated cirrhosis, decompensated cirrhosis, and HBV-related hepatocellular carcinoma (HCC) were included. The average annual hospitalization costs of the above patients were analyzed with SPSS Statistics 20.0 (International Business Machines Corporation, Armonk, New York). And the median and interquartile range were used to indicate the centralized and discrete trends (Table S3).

No liver transplantation history was found in patients hospitalized in the local hospital. Thus, the costs related to transplantation were referred from previous literature (62943 US$ in the first year and 10827 US$ in the subsequent year in China) and calculated with a discount rate of 3%.

Table S3. Hospitalization costs for different states of HBV-related patients

States	Annual hospitalized cost, US$	Interquartile range [Q1, Q2], US$
Acute hepatitis B	1531.8	1318.6 [849.7, 2168.4]
Compensated cirrhosis	1020.9	759.0 [821.1, 1580.0]
Decompensated cirrhosis	3210.0	3071.6 [2054.1, 5125.8]
Hepatocellular carcinoma	2035.8	2801.6 [1049.6, 3851.2]

2.3.2 Outpatient cost

We assumed the outpatient costs for CHB and inactive HBsAg carriers under conventional pattern defaulted 0 because these patients were often unaware of their infection. Once cirrhosis or HCC developed, they went to the hospital and regular follow-up recommend by the guideline would be performed by doctors. The cost consisted of the registration fee, antiviral treatment fee, and examination fee (Table S2). The antiviral drug employed in this study was Tenofovir Disoproxil Fumarate Tablets (Daiwei) produced by Supor South Ocean Pharmaceutical Co., Ltd. (Hangzhou, China), with the specification of 0.5 mg*30 tablets/box and priced US$ 1.6 after the government centralized procurement. Patients with cirrhosis or HCC were followed up every 3 months for blood routine, biochemical function, HBV DNA quantification, HBV serological markers, alpha fetoprotein, liver ultrasonography, and elastography with an annual cost of US$ 231.4.

2.4 Treatment costs

Compared to the conventional pattern, carriers and CHB patients should also undergo regular follow-ups. Immune tolerant individuals and inactive HBsAg carriers were followed up every 6 months for blood routine, biochemical routine, HBV serological

markers, alpha fetoprotein, liver ultrasonography, and elastography, and the average annual cost was US$ 75.7. CHB patients treated with NAs were followed up every 3 months for blood routine, biochemical function, HBV DNA quantification, HBV serological markers, and 6 months for alpha fetoprotein, liver ultrasonography, and elastography with an annual cost of US$ 205.3.

3. Screening intensity assumption

We have carried out HBV screening in our demonstration areas since 2009. The sum of rural residents in Yuhuan County was around 118.9 thousand[1], and 63.9 thousand completed the screening tests annually on average in recent 4 years, with an average annual screening intensity of 53.8%. Additionally, we only performed HBV screening in individuals aged 15-60, thus, the screening intensity was higher than 53.8% in the actual situation. Therefore, we select the base value of 50%. Considered there have been more than 50% of rural residents participating in the screening program, we are exploring the feasibility of expanding the screening coverage to find an optimal intensity that is cost-effective.

Reference:

1. Statistical Yearbook 2020 of Taizhou City. http://tjj.zjtz.gov.cn/. Updated December 4, 2020. Accessed August 1, 2021.
