# Supplementary material for: Cost-Effectiveness of Hepatitis B Mass Screening and Management in High-Prevalent Rural China: A Model Study From 2020 to 2049
Source: Int J Health Policy Manag. 2021 Sep 7;11(10):2115–23. doi: 10.34172/ijhpm.2021.126 (PMC9808295; doi:10.34172/ijhpm.2021.126)
Supplement: Supplementary file 3 — contains Table S5 and Figures S3-S6. [file ijhpm-11-2115-s003.pdf]

Article title: Cost-Effectiveness of Hepatitis B Mass Screening and Management in High-Prevalent Rural China: A Model Study From 2020 to 2049

Journal name: International Journal of Health Policy and Management (IJHPM)

Authors' information: Xiaolan Xu[1]¶, Chensi Wu[1]¶, Lushun Jiang[1], Chunting Peng[1], Liya Pan[1], Xue Zhang[1], Wei Shen[1], Lin Chen[1], Zhuoqi Lou[1], Kaijin Xu[1], Lanjuan Li[1], Yin Dong[2]*, Bing Ruan[1]*

[1]State Key Laboratory for Diagnosis and Treatment of Infectious Diseases, National Clinical Research Center for Infectious Diseases, Collaborative Innovation Center for Diagnosis and Treatment of Infectious Diseases, The First Affiliated Hospital, College of Medicine, Zhejiang University, Hangzhou, China.

[2]People's Hospital Medical Community of Yuhuan County, Taizhou, China.

¶Both authors contributed equally to this paper.

(*Corresponding author: Yin Dong; Email: 9597082@qq.com &

Bing Ruan; Email: ruanbing@zju.edu.cn)

Supplementary file 3. Additional Tables and Figures

Table S5. Incremental cost-effectiveness ratio (ICER) and net monetary benefit (NMB) between intervention strategies for different screening intensities

Screening intensity	Strategy	Average per person		Incremental		ICER, US$/QALY	NMB, US$
		Cost, US$	QALYs	Cost, US$	QALYs		
**50%**	Immunization	318	17.46	-	-	-	40219
	Comprehensive	323	17.58	4.5	0.13	35	40508
	Conventional pattern	341	17.45	18.3	-0.14	dominated	40170
	Treatment	351	17.57	28.7	-0.01	dominated	40454
**60%**	Immunization	316	17.46	-	-	-	40226
	Comprehensive	326	17.61	9.8	0.15	65	40569
	Conventional pattern	341	17.45	15.0	-0.17	dominated	40170
	Treatment	360	17.60	34.4	-0.01	dominated	40503
**70%**	Immunization	317	17.46	-	-	-	40231
	Comprehensive	336	17.64	19.2	0.18	108	40623
	Conventional pattern	341	17.45	5.2	-0.19	dominated	40170
	Treatment	376	17.62	40.1	-0.02	dominated	40547
**80%**	Immunization	321	17.46	-	-	-	40232
	Conventional pattern	341	17.45	20.1	-0.02	dominated	40170

	Comprehensive	356	17.67	35.2	0.20	174	40667
	Treatment	402	17.65	45.9	-0.02	dominated	40579
**90%**	Immunization	332	17.47	-	-	-	40226
	Conventional pattern	341	17.45	9.5	-0.02	dominated	40170
	Comprehensive	394	17.69	62.1	0.23	272	40693
	Treatment	445	17.67	51.6	-0.02	dominated	40595
**100%**	Conventional pattern	341	17.45	-	-	-	40170
	Immunization	352	17.47	11.2	0.02	504	40211
	Comprehensive	459	17.72	106.3	0.25	420	40692
	Treatment	516	17.70	57.3	-0.02	dominated	40583

Willingness-to-pay is US$ 2,322 per QALY gained. QALY, quality-adjusted life-year

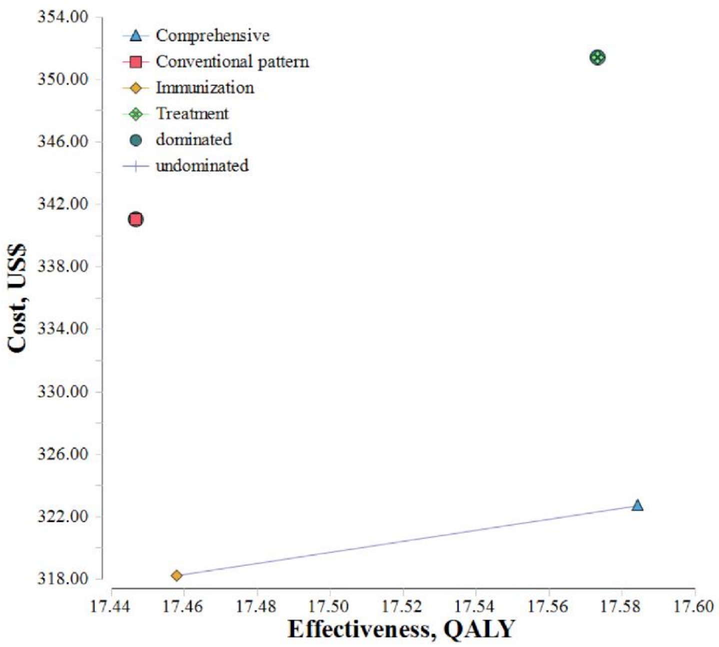

Figure S3. Cost-Effectiveness graph at a screening intensity of 50%. The oblique line is the cost-effective frontier. Strategies on the upper left of the cost-effective frontier were considered dominant for higher cost and lower effect. The willingness-to-pay threshold is US$ 2,322 per QALY gained. QALY, quality-adjusted life-year

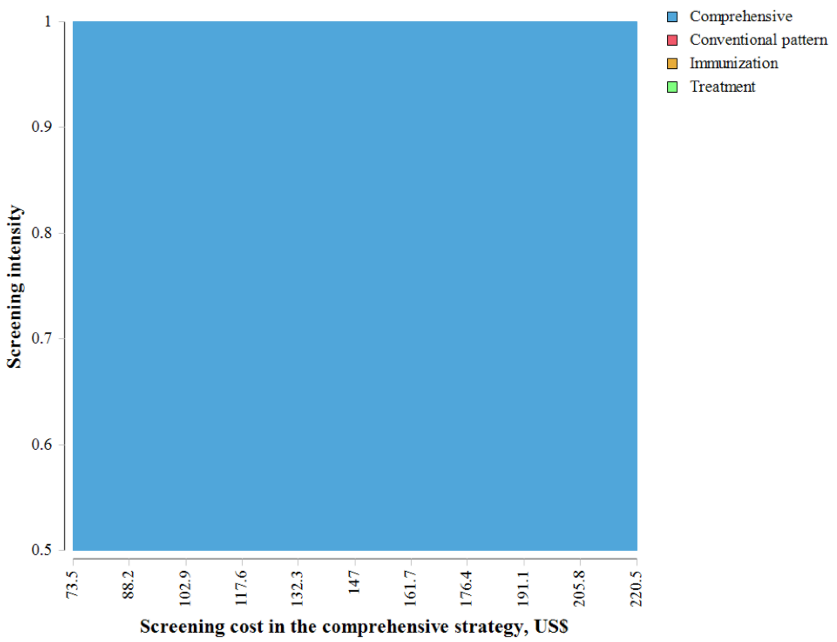

Figure S4. Two-way sensitivity analyses of the screening costs in the comprehensive strategy and screening intensity.

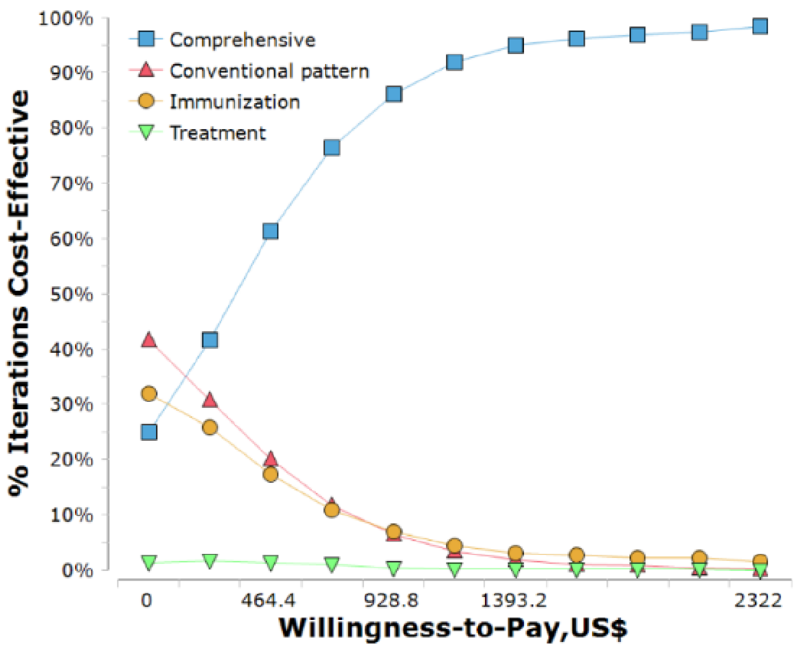

Figure S5. Cost-effectiveness acceptability curve of probability sensitivity analysis.

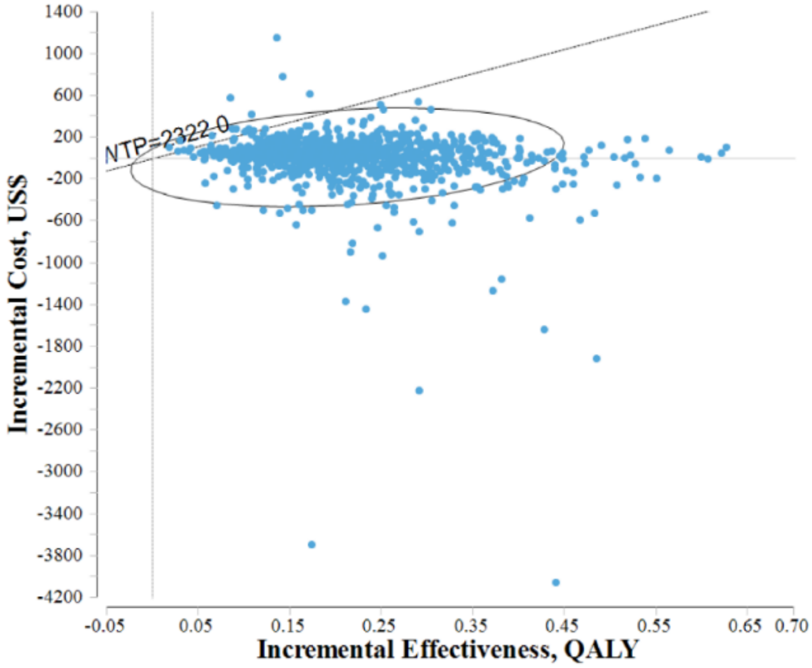

Figure S6. Incremental cost-effectiveness ratio (ICER) scatter plots of probability sensitivity analysis (comprehensive strategy vs conventional pattern). Every blue dot represents a specific individual in the simulating cohort and those below the dashed line are considered cost-effective. QALY, quality-adjusted life-year; WTP, willingness-to-pay
